# In Reply to: CD4+CD8+ Double-Positive T-Lymphocytes: Pitfalls

**DOI:** 10.4274/tjh.galenos.2020.2020.0171

**Published:** 2020-08-28

**Authors:** Miguel Santiago Gonzalez-Mancera, John Mario Gonzalez

**Affiliations:** 1 Universidad de los Andes, School of Medicine, Grupo de Ciencias Básicas Médicas, Bogotá, Colombia

**Keywords:** Double-positive T-lymphocytes, Flow cytometry, Natural killer T-cells

## To the Editor,

It was with great interest that we read the recent reply to our published article. Although the effects of nicotine on the immune response have been described in some regards, we do not know its influence on double-positive T lymphocytes (CD4+CD8+ or DPTs). Our study included volunteers from the Colombian Red Cross who underwent screening as blood donors; however, their smoking status was not evaluated [1].

In the reply to our article, it was advised that the potential phenotypic overlap of natural killer T (NKT) cells with the DPT subpopulation be studied in our cohort. It was mentioned that CD45, a well-known pan-leukocyte marker, could be a possible part of the phenotypic panel. Nonetheless, CD45 would not discriminate between different white cell lineages. NKT cells are a subpopulation of T cells expressing CD16/CD56+ that are CD4+CD8+ double-positive cells during their thymic selection [2]. Therefore, NKT cells that have prematurely escaped from the thymus could explain, to some extent, the presence of NKT double-positive cells in the peripheral blood.

Zloza et al. [3] described 6 different subpopulations of CD3+ T cells according to the intensity of CD4 and CD8 expression. In that study, they also showed the presence of invariant NKT (CD3+CD6B11+) and non-invariant NKT (CD3+CD16/56+) cells as part of DPTs, mainly in the CD4^bright^CD8^dim^ subpopulation. Interestingly, activation-induced expression of CD56 by CD8+ T cells has been described, and it is associated with a reprogramming of the cytolytic activity and cytokine secretion profile in vitro [4]. Furthermore, CD56 is expressed by CD4+ T cells under certain pathological conditions [5]. Due to the complexity of marker expression on these T cell subpopulations, it seems necessary to sort them and to study their gene expression profiles to define specific DPT subpopulations. Nonetheless, it is reasonable to consider that a low percentage of NKT cells could be present in DPTs, but the percentage should be lower than 26% [3].
